# Ketogenic diet in childhood epilepsy: clinical algorithm in a tertiary care center

**DOI:** 10.3389/fped.2023.1221781

**Published:** 2023-07-06

**Authors:** Sanaa Shaaban, Mohammed Al-Beltagi, Omnia El Rashidy, May Nassar, Yasmin El Gendy

**Affiliations:** ^1^ Children's Hospital, Faculty of Medicine, Ain Shams University, Abbassia, Egypt; ^2^ Paediatric Department, Faculty of Medicine, Tanta University, Tanta, Egypt; ^3^ Paediatric Department, University Medical Center, King Abdulla Medical Center, Arabian Gulf University, Dr. Sulaiman Al Habib Medical Group, Manama, Bahrain

**Keywords:** dietary therapy, intractable childhood epilepsy, ketogenic diet algorithm, seizure reduction, treatment

## Abstract

**Background:**

Dietary therapies play a crucial role in managing patients, especially those who have specific types of epilepsy, display adverse effects, or are not responding to pharmacological treatments. The ketogenic diet (KD) is a high-fat, restricted carbohydrate, and adequate protein regimen. The KD has proven to be an effective nonpharmacological treatment for drug-resistant epilepsy (DRE) by generating ketones that act as an alternative fuel source for the brain, thereby reducing the occurrence of seizures. The advantages of KD have been attributed to its universal availability, numerous administration techniques, and affordability.

**Objective:**

This article presents the KD algorithm developed by a multidisciplinary team of experts at the Children's Hospital, Ain Shams University, Egypt. The algorithm serves as a guide for implementing the KD in the treatment of DRE in children. The algorithm has been previously validated through a study.

**Methods:**

The algorithm consists of seven essential stages: (1) referral of patients to the Complex Epilepsy Committee, (2) pre-diet assessment of patients, (3) referral of patients to the Clinical Nutrition (CN) team, (4) diet selection and initiation, (5) seizure follow-up and diet fine-tuning, (6) diet reassessment after 3 months, and (7) evaluation of the KD journey after 24 months.

**Results:**

The KD algorithm was systematically developed and proved highly influential in facilitating the implementation of the KD. The algorithm yielded significant health benefits in pediatric patients.

**Conclusion:**

The KD algorithm provides a systematic approach to implementing the ketogenic diet and has demonstrated positive health outcomes in pediatric patients.

## 1. Introduction

Dietary therapies are a crucial part of treating epilepsy patients, especially those who have specific epilepsy syndromes, display adverse effects, or are not responding to pharmacological treatments, termed drug-resistant epilepsy (DRE) (1, 2). DRE is defined as the failure of adequate trials of two tolerated, appropriately chosen and used antiepileptic drug (AED) schedules (whether as mono-therapies or in combination) to achieve sustained seizure freedom (3).

The ketogenic diet (KD) is a well-established and effective nonpharmacological treatment for DRE and specific epilepsy syndromes. It is a high-fat, restricted carbohydrate, and adequate protein regimen that was first applied in treating epilepsy in the early 1920s (1, 4–6). It was initially developed in the United States, specifically at the Mayo Clinic in Rochester, Minnesota (2, 4, 5). It has gained the attention of academics and the general population as an effective and reasonable solution against epilepsy (7).

The prevalence, epidemiology, and treatment gaps in epilepsy have been the study's focus in Egypt. Published reports indicate that in the largest district of Egypt (New Valley), more than 61% of individuals diagnosed with active epilepsy, including men, women, and children, do not receive any treatment (39%) or are not provided with appropriate treatment (22%) (8). In another study conducted in the Al-Quseir area, there were approximately 111 cases of active epilepsy, including men, women, and children, of which 25% never received any treatment and more than 58% received inaccurate treatment (9). The treatment gap can be primarily attributed to factors such as a shortage of skilled staff, high treatment costs, cultural beliefs, and limited availability of anti-seizure drugs (10).

While the use of the KD as a treatment for drug-resistant epilepsy (DRE) has shown success in various parts of the world, there is limited information on its implementation and utilization in Egypt (11, 12). To raise awareness about KD treatment, a team of expert pediatricians from the Children's Hospital, Faculty of Medicine, Ain Shams University (Egypt), has developed an algorithm for implementing KD as a treatment option. This work aims to review and discuss the development of this algorithm, which can serve as a concise manual for hospitals and healthcare professionals in managing and implementing KD treatment for patients, particularly children, with DRE.

## 2. Methods

**Figure 1:**
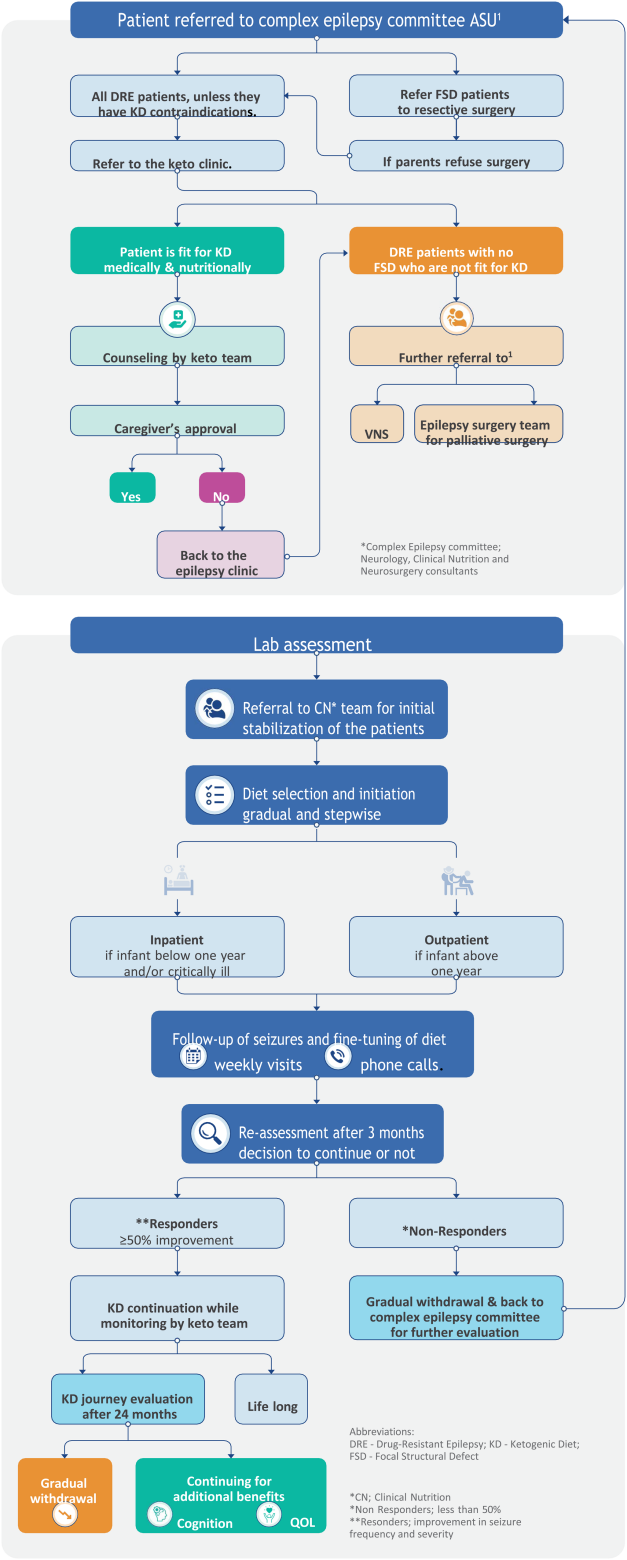
Algorithm for implementing and managing the ketogenic diet in Children's Hospital, Faculty of Medicine, Ain Shams University, Egypt.

The clinical algorithm developed by the multidisciplinary expert faculty is called the ketogenic diet algorithm (KDA) (Figure 1). This algorithm guides medical professionals in making informed decisions and monitoring patients’ progress. It has been previously validated through a study conducted by the same team (13).

The KDA was created by expert clinicians from Ain Shams University Children's Hospital, drawing upon their extensive experience of more than 12 years, opinions, and relevant published papers. The development process involved identifying performance requirements, addressing challenges, and finding suitable solutions. The team conducted a thorough review of the literature and studied published guidelines. Several drafts were created, tested, revised, edited, and refined to ensure the algorithm's accuracy and effectiveness. The resulting clinical algorithm provides a precise sequence of clinical decisions and provisions for appropriate feedback.

### 2.1. Ketogenic Diet Algorithm

The KDA is designed to encompass a series of steps involved in the non-pharmacological treatment of epilepsy, specifically using dietary therapy, primarily focusing on the KD.

Key features of the KDA are as follows:

(A) The KDA consists of seven crucial stages, listed as follows:
  (1) Patient referral to the Complex Epilepsy Committee.
  (2) Pre-diet assessment of patients.
  (3) Patient referral to the Clinical Nutrition (CN) team.
  (4) Diet selection and initiation.
  (5) Follow-up of seizures and fine-tuning of diets.
  (6) Reassessment of the diet after 3 months.
  (7) Evaluation of the KD journey after 24 months.
(B) The KDA is designed as a systematic and logical tool, providing valuable assistance in managing different KD treatment protocols. Furthermore, it offers alternative options at critical branching points, ensuring flexibility and tailored approaches to patient care.

By incorporating these salient features, the KDA is a comprehensive and practical resource for healthcare professionals, facilitating the effective implementation and management of KD treatment for epilepsy.

In the initial evaluation phase, patients with epilepsy were assessed to determine their suitability for other medical or surgical management options in cases with focal structured defect (FSD), including curative resective epilepsy surgery to be performed. If surgery was not deemed appropriate or the family refused, the KD therapy recommendation was followed. The implementation and management of the KD is a complex process that requires the collaborative efforts of a multidisciplinary team of experts.

At the Children's Hospital, Ain Shams University (Egypt), the core team responsible for overseeing the process is known as the Complex Epilepsy Committee. This team consists of expert consultants from the neurology and clinical nutrition departments, with additional input from neurosurgery consultants for patient enrollment. In cases where a patient was found unsuitable for KD, the expert team provided further guidance and advice based on the patient's condition, referring them back to the committee for vagal nervous stimulation (VNS) or palliative epilepsy surgery.

Once a patient was enrolled in the KD program, a specialized nurse, psychologist, nutritional educator, and dedicated chef joined the team to provide their expertise. This approach aligns with previous reports, which recommend the involvement of a neurologist experienced in KD treatment, a skilled pediatric nutritionist, a specialist nurse, a nutritional educator, a social worker, and a chef knowledgeable in ketogenic diets (6, 14–17). The team collectively aimed to achieve several primary goals, including assessing patients’ suitability for KD treatment, providing continuous care and monitoring throughout the KD journey, counseling parents on KD principles, assisting with diet implementation and meal preparation plans, monitoring patient progress, and addressing any complications that may arise from being on the ketogenic diet (15, 16).

#### 2.1.1. Selection of patients

The algorithm begins with the first critical stage, which involves referring the patient to the Complex Epilepsy Committee at the Faculty of Medicine, Ain Shams University (Egypt). During the evaluation process, the committee confirms the diagnosis of drug-resistant epilepsy (DRE) and identifies the specific type of seizures experienced by the patient, such as focal with awareness, Focal with impaired awareness, motor onset, generalized epilepsy where epileptic spasm tonic-clonic, a tonic or Myoclonic and hyperkinetic motor seizure (18). Based on the assessment, patients with focal structured defects suitable for curative surgery are referred to the neurosurgery department. In contrast, those who are unsuitable or whose family refused surgery are offered the option of KD treatment.

The KD is chosen as the treatment for children diagnosed with DRE who are not eligible for curative surgery and fall within the age range of 0–18 years. Before being directed to the Keto Clinic, all patients approved for KD treatment by the Keto team undergo screening to ensure they do not have any contraindication for KD as fat metabolism-related disorders (such as β-oxidation defects and medium-chain 3-hydroxy acyl-CoA deficiency) or other conditions like porphyria. This screening is crucial as the KD primarily involves a high-fat diet, and patients with underlying fatty acid metabolism disorders placed on the KD may experience a potentially severe catabolic crisis (2).

Table 1 lists the contraindications (2, 6) that exclude KD as a treatment option for certain DRE patients. It helps identify cases where the KD may not be suitable for implementation.

**Table 1:** Contraindications for the KD treatment.

Absolute contraindications for the KD	Relative contraindications for the KD
Carnitine deficiency (primary)	The presence of surgical focus as being identified by neuroimaging and video electroencephalogram monitoring
Carnitine palmitoyl-transferase I or II deficiency	Inability to maintain adequate nutrition (fastidious eater)
Carnitine translocase deficiency	Parent or caregiver non-compliance
Pyruvate carboxylase deficiency	Propofol concurrent use (risk of propofol infusion syndrome may be higher)
Porphyria	
β-oxidation defects: Short-chain acyl dehydrogenase deficiency Medium-chain acyl dehydrogenase deficiency Long-chain acyl dehydrogenase deficiency Medium-chain 3-hydroxy acyl-CoA deficiency Long-chain 3-hydroxy acyl-CoA deficiency	

#### 2.1.2. Pre-diet assessment of patients at the Keto clinic

At the Keto Clinic, all patients underwent various evaluation programs to ensure their medical and nutritional suitability for the KD treatment, following the guidelines set by the algorithm. These evaluation programs included metabolic, nutritional, and laboratory tests, which were carried out to gather a comprehensive medical profile and assess the patient's health status before initiating the treatment.

##### 2.1.2.1. Metabolic evaluation

The patients underwent assessments to identify and exclude any metabolic disorders that could be contraindications for the diet (as outlined in Table 1). Additionally, the evaluation aimed to identify any pre-existing complications associated with the diet, which needed to be addressed before starting the treatment. Examples of such complications included liver disease, dyslipidemia, kidney stones or gravel, gastroesophageal reflux, constipation, cardiomyopathy, poor oral intake, and faltering growth (2).

##### 2.1.2.2. Nutritional evaluation

The nutritional status of the patients was assessed by analyzing their physical parameters, such as weight, height/length, ideal weight for their stature, and body mass index. These assessments were conducted following the guidelines for KD treatment, which have been previously published in peer-reviewed journals (2, 7, 15, 16).

The Keto team collected a week's worth of dietary information from each patient, considering their food preferences, allergies, aversions, and intolerances. This comprehensive history-gathering process encompassed various diet formulations, including milk-only for infants, oral or enteral (tube feeding), or a combination (6). Based on each patient's dietary history and nutritional profile, specific dietary requirements were calculated, including energy level, ketogenic ratio, proteins, and fluid level. Additionally, appropriate amounts of minerals and vitamins were supplemented as necessary. This personalized approach to dietary plans (Section 3.4) played a crucial role in developing individualized diets that catered to each child's and their family's unique needs rather than relying solely on the perceived effectiveness of different KD types (5, 15, 16, 19).

##### 2.1.2.3. Laboratory evaluation

Several routine laboratory tests were conducted to gain insights into the patient's health. These tests encompassed a complete blood count with platelets, electrolyte analysis (serum bicarbonate, total protein, calcium, magnesium, phosphates, zinc, and selenium), liver and kidney function tests (albumin, alanine aminotransferase, aspartate aminotransferase, blood urea nitrogen, and creatinine levels), fasting lipid profile, and urinalysis, including urine calcium and creatinine (20). If applicable, special attention was given to the calcium–creatinine ratio (21) and the presence and levels of anti-seizure Medications (ASM) drugs (2, 22).

Furthermore, specific comprehensive tests were conducted to assess the metabolic profile of the patients. These included blood ammonia and lactate levels, evaluation of cerebrospinal fluid (glucose, protein, lactate, and glycine), serum acylcarnitine profile, serum amino acids, and urine organic acids. Optional tests were also prescribed in cases where patients had pre-existing medical conditions. For instance, a renal ultrasound and nephrology consultation were recommended for patients with a history of kidney stones, recurrent urinary tract infections, or those taking topiramate or zonisamide. Additionally, an electrocardiogram was performed for patients with a history of heart disease (2).

An extensive literature survey highlighted the importance and significance of conducting comprehensive testing (2, 19, 22). For example, urinalysis with urine calcium-to-creatinine ratio tests played a crucial role in identifying elevated ratios, indicating a potential risk for kidney stone formation. Potential complications like these could be identified by conducting pre-diet evaluation and testing, enabling proactive steps to mitigate them (19, 21). Genetic sequencing to be performed specially for patients with progressive epileptic encephalopathy (2).

Patients who were deemed medically and nutritionally unfit for KD treatment were referred back to the Keto team (Complex Epilepsy Committee), with the option of considering vagal nerve stimulation therapy and/or palliative surgical treatment (23).

##### 2.1.2.4. Counseling and consent

The subsequent stage of the algorithm involved counseling the parents of patients who were deemed suitable candidates for KD treatment. The Keto team conducted individual counseling sessions with each patient and his family using the data gathered from the pre-diet evaluations (metabolic, nutritional, and laboratory). These sessions provided crucial information about the principles of KD and their role in meal preparation, diet maintenance, and monitoring ketosis. The significance of strict adherence to the diet and the need for vitamin and mineral supplementation were emphasized (2, 6). Following the recommended dosing guidelines from the literature, parents were taught how to calculate and weigh ingredients for meal preparation (2, 6). Additionally, using seizure diaries to track seizure frequency and duration (19) and video recording whenever possible were encouraged. Further reading of Arabic brochures, prepared and provided at the Children's Hospital, Faculty of Medicine, Ain Shams University, based on the literature review, was also recommended. Moreover, in-depth discussions were held regarding the neurological aspects of KD, including seizure reduction, cognitive expectations, psychosocial issues related to KD, tolerability (potential side effects of the diet), and medication. Other topics addressed included expectations during hospital admission or minor illness, the need for ongoing ketone monitoring, and regular outpatient follow-up (2).

After the counseling sessions were completed, the subsequent step in the algorithm involved seeking guardian approval. The parents or caregivers of the patients were asked to provide informed and written consent. With their authorization, the patients could proceed with KD initiation. Patients whose parents rejected or withheld consent for the proposed KD treatment were referred back to the Complex Epilepsy Committee to explore alternative non-pharmacological treatment options.

##### 2.1.2.5. Laboratory assessment

After obtaining parental approval, the next crucial step in the KD algorithm (KDA) entailed a comprehensive review of the laboratory evaluations by the Keto team. The tests conducted during the pre-diet evaluation phase (Section 3.2.3) were thoroughly examined, and additional tests were prescribed if necessary. This meticulous process ensured that all aspects of the patient's medical profiles were reviewed, updated, and deemed appropriate before proceeding to the subsequent critical stages.

#### 2.1.3. Referral to the clinical nutrition team

Upon successfully completing the final inspection, the patients were referred to the Clinical Nutrition (CN) team, marking the transition to the next crucial stage of the algorithm. The CN team was responsible for stabilizing the patients and preparing them for diet selection. The patient data were thoroughly reviewed to develop a personalized nutritional plan that would address any existing malnutrition or undernutrition conditions before the initiation of the KD treatment. This step ensured that the patient’s nutritional needs were adequately met and optimized before commencing the KD.

#### 2.1.4. Diet selection

Diet formulation, selection, and construction were based on the specific circumstances of each child and their family rather than solely relying on the perceived efficiency of a particular type of KD. This approach allowed for customizing diets that best suited the individual patient, thereby achieving optimal treatment conditions.

**Figure 2:**
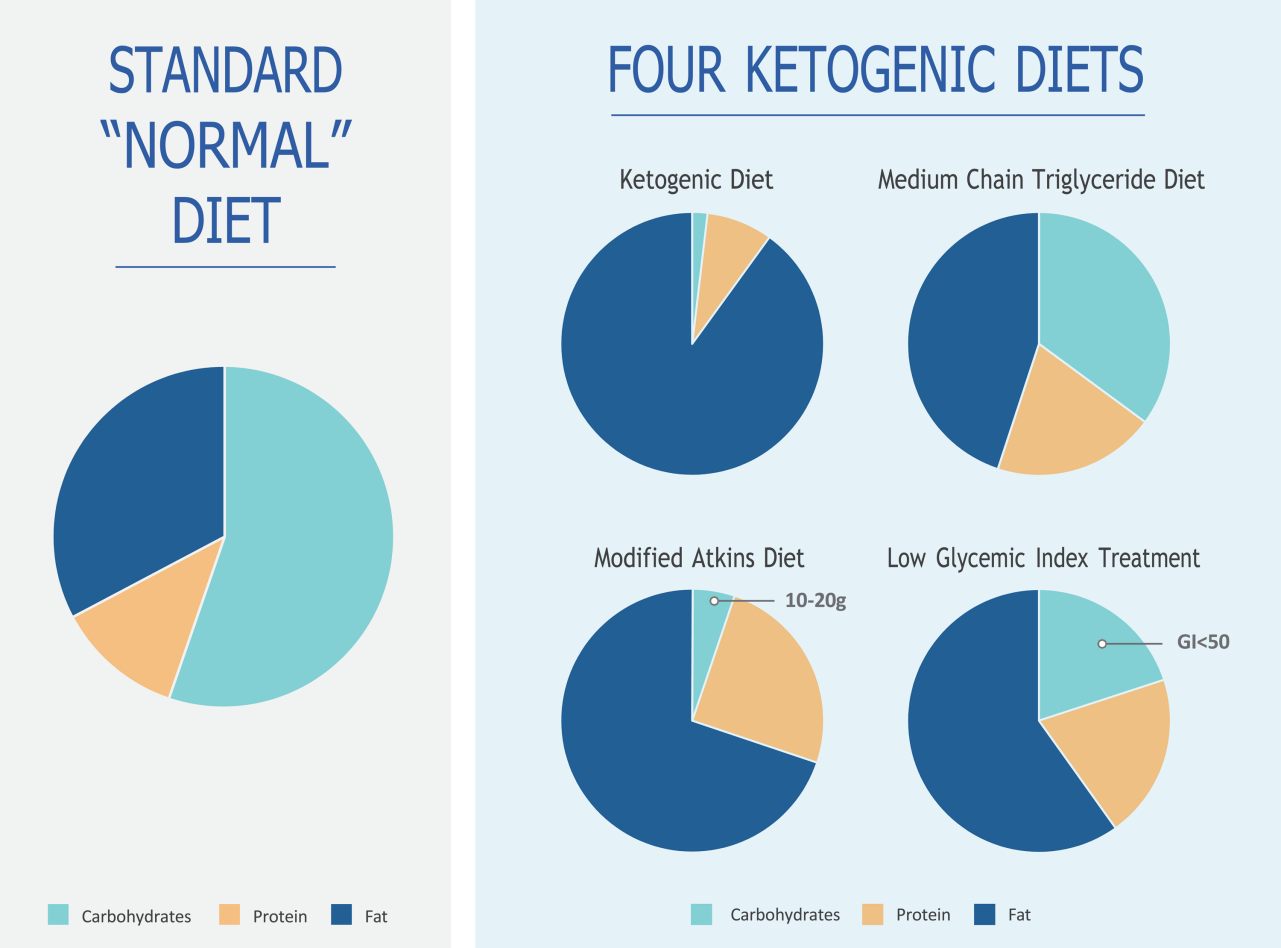
The four types of ketogenic diets utilized.

The decision regarding the type of KD to be implemented was made from four main types of diets (Figure 2), namely, the classic KD, medium-chain triglyceride (MCT) KD, modified Atkins diet (MAD), and low glycemic index treatment (LGIT) (2). In certain situations, a hybrid KD known as the modified ketogenic diet (MKD) was utilized, incorporating principles from one or more of the main KD regimens. This approach allowed the development of new features tailored to patients’ needs, balancing the required ketosis and palatability. Currently, the majority of KDs are, in fact, individualized MKDs, with each diet differing from one another but tailored to the specific requirements of each patient (24, 25).

#### 2.1.5. Classic KD

The primary source of fat in the KD is predominantly derived from long-chain triglycerides obtained from standard traditional foods. There are no specific restrictions on calorie intake or fluid consumption (2). For infants and children who are fed through gastrostomy and jejunostomy tubes, an all-liquid, formula-based KD can be utilized, which has shown high compliance and efficacy rates. The classic KD is typically employed for children over the age of two or those who are tube-fed (6). The most common ratio used for children above two years of age is 4:1, whereas a ratio of 3:1 or lower is used for infants. In the case of infants, adjustments may be made to increase either the protein or carbohydrate content to ensure optimal growth and development. This protocol aligns with established research findings (2, 6, 15, 16). Breastfeeding is allowed by incorporating the amount of breast milk into a 3:1 formula or through brief feedings on demand.

#### 2.1.6. Medium-chain triglyceride KD

The MCT KD is distinguished by its higher carbohydrate and protein content compared to the classic KD. This is because MCT oils offer a higher yield of ketones (per kilocalorie of energy) and are better absorbed by the liver. As a result, less fat is required in the diet, allowing for increased quantities of carbohydrates and proteins (2, 6, 15, 16).

At the Children's Hospital, a modified MCT KD was implemented using 30% MCT oil and, if necessary, 30% long-chain fats. This approach was chosen to address previous reports of gastrointestinal discomfort associated with a 60% MCT composition. MCT oil was administered in the diet as coconut oil or emulsion (2, 6). The benefits of MCT oil have been well-documented in the literature. It supplements the classic KD, promoting ketosis, improving lipid abnormalities, and aiding in bowel movement. Better tolerability was achieved by providing smaller amounts of MCT with each meal but increasing the frequency of meals (2).

#### 2.1.7. Modified Atkins diet

The MAD is a high-fat, low-carbohydrate therapy like the classic KD regarding food choices. It does not restrict protein, fluid, or calorie intake (2, 5). The MAD is typically administered in an outpatient setting, making it time-efficient and allowing for greater parental independence as it does not require precise weighing of food ingredients (2, 11).

The MAD generally provides a ketogenic ratio ranging from 1:1 to 1.5:1, although no fixed ratio is mandated. Initially, the daily carbohydrate consumption on the MAD is typically around 10–15 g, which is comparable to the strict initiation phase of the Atkins diet used for weight loss. After 1–3 months, there may be a possibility of increasing the daily carbohydrate intake to 20 g (2, 6).

#### 2.1.8. Low glycemic index treatment

The LGIT diet allows for a higher daily intake of carbohydrates, ranging from 40 to 60 g per day. However, there is strict regulation regarding the type of carbohydrates consumed, with a preference for those that have minimal impact on blood glucose levels, such as carbohydrates with low glycemic indexes below 50 (6). Like the MAD, the LGIT diet does not require precise weighing of food ingredients and is known to require less time for meal calculations, offering more parental independence (2). It is worth noting that at the Keto Clinic, the classic KD therapy is typically offered to patients (7), while the MAD is utilized when the classic KD is challenging for families to follow (11). The MCT diet is less popular among patients at this Keto Clinic. The LGIT diet is individually tailored and is often the preferred choice for teenagers.

##### 2.1.8.1. Calculation of dietary requirements

Before starting a diet, the last step is to calculate each patient's specific dietary needs, such as their ketogenic ratio, protein levels, and fluid intake, tailored to their diet plan.

i. Assessment of calorie requirements

Determining calorie requirements for each person is crucial to ensure healthy growth and development based on their age (as illustrated in Table 2). We calculate the ideal body weight (IBW) appropriate for their age to achieve this. For children aged 0–2 years, IBW is determined by their weight at the 50th percentile for their length. For those aged 2–18 years, IBW is calculated using the weight corresponding to the 50th percentile body mass index minus the ideal body mass index×(height, in meters)^2^ (27, 28).

**Table 2:** Energy requirements for the patients of different age groups [adapted from Butte (26)].

	2002 Institute of Medicine		22004 FAO/WHO/UNU			
Age, years	MJ/day	kcal/day	MJ/day	kcal/day	kJ/kg/day	kcal/kg/day
1–2	3.9	930	4.0	950	345	82
2–3	4.7	1,120	4.7	1,125	350	84
3–4	6.2	1,485	5.2	1,250	334	80
4–5	6.6	1,566	5.7	1,350	322	77
5–6	6.9	1,658	6.1	1,475	312	74
6–7	7.3	1,742	6.6	1,575	303	73
7–8	7.7	1,840	7.1	1,700	295	71
8–9	8.1	1,931	7.7	1,825	287	69
9–10	8.550	2,043	8.3	1,975	279	67
10–11	9.0	2.149	9.0	2,150	270	65
11–12	9.5	2,279	9.8	2,350	261	62
12–13	10.2	2,428	10.7	2,550	252	60
13–14	11.0	2,618	11.6	2,775	242	58
14–15	11.8	2,829	12.5	2,000	233	56
15–16	12.6	3,013	13.3	3,175	224	53
16–17	13.2	3,152	13.9	3,325	216	52
17–18	13.5	3,226	14.3	3.400	210	50

ii Assessment of protein requirements
  The standard advice for the classic KD is 1 g protein/body weight for older children and 1.5 g/kg for rapidly growing younger children (29). The following protein proportions were implemented based on the age group of the patients:
  ✓ Age 0–1 year (infants): 1.5 g/kg/day,
  ✓ Age 1–3 years: 1.1 g/kg/day,
  ✓ Age 4–13 years: 0.95 g/kg/day,
  ✓ Age 14–18 years: 0.85 g/kg/day, and
  ✓ Age 18 years and above (adults): 0.8 g/kg/day.
iii. Assessment of fluid requirements
  To calculate the necessary fluid intake, we followed the Holliday–Segar method. Based on a weight of 20 kg, we determined that around 1500 ml of fluid was required to replace lost water and address renal loss. For every additional kilogram above 20 kg, an additional 20 ml of fluid was needed (30).
iv Determination of the appropriate ketogenic ratio

The ketogenic ratio was calculated as grams of fat to the sum of grams of protein and carbohydrate, represented as follows:

*Ketogenic ratio = fat (g): protein (g) + carbohydrates (g)*

The typical ketogenic ratio used in the Keto Clinic ranges from 2:1 to 4:1 (14). However, it was found that individuals may respond differently to the same ratio (15, 16). Infants and adolescents were generally prescribed a lower ratio of 3:1, while children were set on a higher ratio of 4:1 to achieve desired levels of urinary ketones or serum β-hydroxybutyrate (>2,000 mmol/m or >3 mmol/L) (15, 16).

Due to the restriction of fruits, vegetables, and other foods in the ketogenic diet, special attention was given to supplementing the diet with appropriate quantities of minerals and vitamins (5, 15, 16, 18). All patients were prescribed sugar-free multivitamins with minerals, including trace minerals like selenium (15, 16, 31). Calcium supplementation, which met the daily recommended dietary allowance requirements and vitamin D above the recommended dietary allowance, was also provided (6). L-carnitine supplementation was included as well. Oral citrates were prescribed to prevent the formation of kidney stones, particularly in hot weather conditions prevalent in Egypt (5, 19, 21). Optional supplements such as laxatives (e.g., MiraLAX, mineral oil, glycerin suppository), MCT oil, or coconut oil as a source of MCT were also listed in the literature (2).

Prior to initiating the diet, the Keto team reviewed the anticonvulsants and other medications used by the patients to determine their carbohydrate content (2). It was ensured that all drugs were switched to tablet or sugar-free forms approximately two weeks before starting the diet. This step was crucial in eliminating external sources of carbohydrates and facilitating the induction of ketosis (6). Parents/caregivers were also advised to check the carbohydrate content of any prescribed medications or supplements, including over-the-counter ones, for patients on the ketogenic diet.

##### 2.1.8.2. Diet initiation

According to the algorithm, diet initiation was done gradually and stepwise, without fasting. Studies have shown that the no-fasting initiation method reduces the incidence of complications associated with the ketogenic diet (18, 32). However, it should be noted that fasting can be beneficial in cases where an immediate response is desired for rapid seizure reduction (2). Fasting was not utilized in the Children's Hospital, Ain Shams University, Egypt.

For infants below 1 year or who need special medical care, the classic KD was initiated in a hospital setting to ensure close observation of the child and prompt medical intervention if any complications arose. This approach allowed caregivers to receive training on food calculation and weighing, ketosis monitoring, and KD management before the patient was discharged from the hospital. Parents and caregivers were also taught about the symptoms and management of acidosis and hypoglycemia (2). Alternatively, the classic KD could be initiated in an outpatient setting if the child is above one year or need no medical care. Families were provided with all the necessary medical information for continuing the diet, and continuous support was made available as recommended (2, 6). Telemedicine was also emphasized, especially during the COVID-19 era when lockdown measures were in place in many countries (33). The MAD was typically initiated in an outpatient setting without a fasting period. In the hospital, all types of KDs, except for patients with critical conditions and children under 1 year of age, were successfully initiated in an outpatient setting.

#### 2.1.9. Follow-up and fine-tuning

During the early stages of diet initiation, the algorithm included regular follow-up visits and diet reassessments as the final critical stages. After discharge, patients were initially seen every week for the first month and then had monthly follow-ups, particularly if expected urinary ketosis was not maintained. These frequent follow-ups allowed for tracking seizure reductions and making necessary diet adjustments. The Keto team maintained continuous communication with patients through platforms like WhatsApp and provided round-the-clock telephone support. Under optimal conditions, follow-up visits were scheduled every 3 months during the first year (2, 34).

During each follow-up visit, nutritional and laboratory evaluations were conducted to monitor the progress of the ketogenic diet in treating drug resistant epilepsy. Blood ketones were checked to ensure the diet's efficacy and compliance. Patients taking carbonic anhydrase inhibitors were closely monitored for an increased risk of kidney stones, considering that the ketogenic diet may exacerbate pre-existing metabolic acidosis, especially in the early stages of diet initiation. Blood pH and bicarbonate levels were monitored, and if a patient experienced symptoms such as vomiting or lethargy, bicarbonate supplements were initiated. Additionally, dietary requirements and supplement levels were reviewed, compliance with therapy was assessed, and minor adjustments were made to improve compliance and fine-tune the diets (2, 6, 7).

#### 2.1.10. Diet reassessment

According to the algorithm, the ketogenic diet was reassessed after 3 months of initiation. Research has shown that most patients (75%) responded to the diet within 14 days, while some took longer, up to 8–10 weeks. Nonresponders, who had less than 50% seizure reduction, were recommended for diet discontinuation and gradually tapered off the ketogenic diet. They were then referred back to the Keto team for further evaluation. Responders, who had more than 50% seizure reduction, continued the diet for 2 years if it was deemed completely successful in reducing seizures (2).

Patients were regularly followed up during the 2-year treatment period to monitor their growth patterns, dietary regimens, and laboratory parameters. If the ketogenic diet was 100% successful, patients who were on anti seizure medication maybe cautiously weaned off them after 6–12 months. The reduction of medications such as phenobarbital and benzodiazepines was closely monitored to avoid seizure exacerbation (6).

For patients with glucose transporter protein type 1 deficiency or pyruvate dehydrogenase deficiency, the duration of the ketogenic diet study was extended. These conditions involve impairments in brain energy metabolism, and the ketogenic diet bypasses these metabolic barriers by providing ketones as an alternative energy source for the brain. Significant results have also been observed in other disorders such as tuberous sclerosis complex, infantile spasms, complex 1 mitochondrial deficiency disorder, Doose syndrome (epilepsy with myoclonic-atonic seizures), febrile infection-related epilepsy syndrome, formula-fed infants, Ohtahara syndrome, and super-refractory status epilepticus (5, 6).

#### 2.1.11. Evaluation after 24 months

At the end of the algorithm, the Complex Epilepsy Committee evaluated the outcomes of successful cases and decided whether to discontinue the diet after 2 years or continue it indefinitely. Nevertheless, patients who wished to extend the diet beyond the initial 2-year period for additional cognitive benefits and improved quality of life were provided with that option.

##### 2.1.11.1. Diet discontinuation

Before discontinuing the diet in seizure-free children, a routine electroencephalogram and a review of clinical data were conducted. This allowed for counseling of families regarding the risk of seizure recurrence, which was estimated to be around 20%. However, studies on this topic indicated that 80% of individuals who had achieved seizure freedom on the ketogenic diet would remain seizure-free even after diet discontinuation (6). Additionally, laboratory assessments were performed to check blood count, lipid profile, and liver and kidney functions.

The process of diet discontinuation involved a gradual tapering off, resembling the weaning off of anti-seizure medications, thereby minimizing the risk of increased seizures. Ideally, the diet should be discontinued for 1 year unless urgent discontinuation is necessary. The weaning process (over a period of 2–3 months) involved gradually lowering the ketogenic ratio from 4:1 to 3:1 to 2:1. Following this, the ketogenic foods were continued while increasing calorie and fluid intake. High-carbohydrate foods were reintroduced only after urinary ketosis was lost (2, 15, 16).

## 3. Anticipated results

Implementing the KDA in our hospital has proven effective in identifying children who can benefit from the ketogenic diet and those who may not. The KDA offers a personalized and individualized nutrition plan tailored to specific needs, considering factors such as age, weight, height, gender, activity level, and overall health status. With the KDA, accurate calculations of macronutrient ratios and caloric intake can be made, ensuring that the ketogenic diet provides the desired amounts of carbohydrates, proteins, and fats for each patient. This standardized approach allows for easy accessibility to a well-defined ketogenic diet for all patients.

Moreover, the KDA facilitates strict monitoring and tracking of a patient's condition, food intake, and macronutrient ratios. This level of monitoring enhances compliance and adherence to the diet, which, in turn, leads to improved outcomes. By following a personalized KDA, individuals may experience faster epilepsy control and improvements in other health markers. Additionally, the KDA helps prevent nutrient deficiencies and ensures that patients meet their daily nutritional requirements.

Furthermore, the KDA simplifies the management of complex ketogenic diet treatment modules, making it easier for junior healthcare professionals to administer. It also aids in the early detection of potential complications and provides alternative options when necessary. Implementing the KDA has also contributed to cost reduction, minimizing long-term food and healthcare expenses while reducing the risk of chronic diseases such as diabetes and heart disease, which come with their costs.

Finally, the KDA enables the collection and analysis of data on individuals’ progress and outcomes, offering valuable insights into the effectiveness of the ketogenic diet and identifying areas for improvement or optimization. It is important to note that the efficacy of the KDA may vary from person to person. For more information, please refer to our previously published work on using the DKA (13).

## 4. Discussion

Applying the KDA in patients with drug resistant epilepsy holds promise as an approach to ensure the precise and personalized implementation of the ketogenic diet, thereby enhancing the likelihood of success in reducing seizure frequency and severity. One notable advantage of the ketogenic diet is its universal availability at affordable prices compared to novel anti-seizure medications (4). Furthermore, the ketogenic diet can be administered through various valid methods (2, 7). Research has demonstrated that the ketogenic diet can yield a more than 90% reduction in seizure frequency for one-third of patients, regardless of the age group, underlying etiology, or seizure type (1).

Algorithms, in general, play a significant role in managing medical disorders by assisting healthcare professionals in making precise and efficient diagnoses, developing customized treatment plans, and monitoring patient progress over time (34). For patients with drug resistant epilepsy following a ketogenic diet, closely monitoring their ketosis state is crucial to prevent potential adverse effects. An algorithm designed to regulate the implementation of the diet can facilitate the tracking of nutrient intake, blood ketone levels, and other essential parameters, ensuring the safety and effectiveness of the diet (26). In our previous studies, the KDA has proven to be a valuable tool in making the ketogenic diet a tolerable, safe, and effective therapy for children with drug-resistant epilepsy without significantly impacting their anthropometric measurements or lipid profiles (7, 11, 13). Developing a flexible algorithm is preferable to previous fixed ketogenic diet protocols, such as restrictive protocols and prolonged fasting (35). Following a well-defined algorithm has been associated with an increased success rate in treating drug resistant epilepsy with the ketogenic diet. Early protocols for the ketogenic diet resulted in a success rate of approximately 25% in treating drug resistant refractory epilepsy in children (36), which increased to 56% after implementing a well-defined algorithm (7, 11, 13).

### 4.1. Limitations of the KDA

While the KDA has demonstrated promise in managing drug resistant epilepsy, it is essential to acknowledge its limitations. Strict adherence to the ketogenic diet can be challenging for certain patients, particularly children or individuals with specific dietary restrictions or food preferences, making it difficult to maintain the prescribed macronutrient ratios. Nutritional deficiencies are common complications, mainly when strict adherence to the diet is not achieved. Side effects such as constipation, nausea, vomiting, and diarrhea may also occur. It is important to note that our experience with the DKA is based on a single-center setting, representing a significant limitation. Further research and multicenter studies are needed to expand our understanding and validate the effectiveness of the KDA across different clinical settings.

## 5. Conclusion

The limited information available on the use and implementation of the ketogenic diet (KD) as a treatment for drug-resistant epilepsy (DRE) in low-resource countries like Egypt prompted the development of a clinical algorithm. Expert faculty members collaborated to formulate the KDA, which provides a precise sequence of clinical decisions and offers alternative pathways to guide medical professionals in decision-making and monitoring patients’ progress. The KDA was successfully implemented at the Children's Hospital, Faculty of Medicine, Ain Shams University, Egypt, and has played a significant role in simplifying the implementation of the ketogenic diet, making it more accessible and manageable for medical professionals.

## Data Availability

The original contributions presented in the study are included in the article/**Supplementary Material**, further inquiries can be directed to the corresponding author.

## References

[1] Henderson CB Filloux FM Alder SC Lyon JL Caplin DA. Efficacy of the ketogenic diet as a treatment option for epilepsy: meta-analysis. J Child Neurol. (2006) 21:193–8. 10.2310/7010.2006.00044 16901419

[2] Kossoff EH Zupec-Kania BA Amark PE Ballaban-Gil KR Christina Bergqvist AG Blackford R Optimal clinical management of children receiving the ketogenic diet: recommendations of the International Ketogenic Diet Study Group. Epilepsia. (2009) 50:304–17. 10.1111/j.1528-1167.2008.01765.x 18823325

[3] Fattorusso A Matricardi S Mencaroni E Dell'Isola GB Di Cara G Striano P the pharmacoresistant epilepsy: an overview on existant and new emerging therapies. Front Neurol. (2021) 12:674483. 10.3389/fneur.2021.674483 34239494 PMC8258148

[4] Kossoff EH McGrogan JR. Worldwide use of the ketogenic diet. Epilepsia. (2005) 46:280–9. 10.1111/j.0013-9580.2005.42704.x 15679509

[5] Freeman JM Kossoff EH Hartman AL. The ketogenic diet: one decade later. Pediatrics. (2007) 119:535–43. 10.1542/peds.2006-2447 17332207

[6] Kossoff EH Zupec-Kania BA Auvin S Ballaban-Gil KR Christina Bergqvist AG Blackford R Optimal clinical management of children receiving dietary therapies for epilepsy: updated recommendations of the International Ketogenic Diet Study Group. Epilepsia Open. (2018) 3:175–92. 10.1002/epi4.12225 29881797 PMC5983110

[7] El-Rashidy OF Nassar MF Abdel-Hamid IA Shatla RH Abdel-Hamid MH Gabr SS Modified atkins diet vs classic ketogenic formula in intractable epilepsy. Acta Neurol Scand. (2013) 128:402–8. 10.1111/ane.12137 23679058

[8] El-Tallawy HN Farghaly WM Shehata GA Abdel-Hakeem NM Rageh TA Abo-Elftoh NA Epidemiology of epilepsy in new valley governorate, Al Kharga district, Egypt. Epilepsy Res. (2013) 104:167–74. 10.1016/j.eplepsyres.2012.08.010 22981337

[9] El-Tallawy HN Farghaly WM Rageh TA Shehata GA Metwally NA Badry R Spectrum of epilepsy–prevalence, impact, and treatment gap: an epidemiological study from Al-Quseir, Egypt. Neuropsychiatr Dis Treat. (2016) 12:1111. 10.2147/NDT.S87765 27257380 PMC4874633

[10] Mbuba CK Ngugi AK Newton CR Carter JA. The epilepsy treatment gap in developing countries: a systematic review of the magnitude, causes, and intervention strategies. Epilepsia. (2008) 49:1491–503. 10.1111/j.1528-1167.2008.01693.x 18557778 PMC3573323

[11] El Rashidy OF Nassar MF El Gendy YG Deifalla SM Gaballa S. Experience with MAD on children with epilepsy in Egypt after classic KD failure. Acta Neurol Scand. (2018) 137:195–8. 10.1111/ane.12856 29034969

[12] Gerges M Selim L Girgis M El Ghannam A Abdelghaffar H El-Ayadi A. Implementation of ketogenic diet in children with drug-resistant epilepsy in a medium resources setting: Egyptian experience. Epilepsy Behav Case Rep. (2019) 11:35–8. 10.1016/j.ebcr.2018.11.001 30619711 PMC6312833

[13] El-Rashidy OF Nassar MF Shokair WA El Gendy YGA. Ketogenic diet for epilepsy control and enhancement in adaptive behavior. Sci Rep. (2023 Feb 6) 13(1):2102. 10.1038/s41598-023-27373-1 36747012 PMC9902473

[14] Hartman AL Vining EP. Clinical aspects of the ketogenic diet. Epilepsia. (2007) 48:31–42. 10.1111/j.1528-1167.2007.00914.x 17241206

[15] Ruan Y Chen L She D Chung Y Ge L Han L. Ketogenic diet for epilepsy: an overview of systematic review and meta-analysis. Eur J Clin Nutr. (2022) 76(9):1234–44. 10.1038/s41430-021-01060-8 35027683

[16] Kim SH Shaw A Blackford R Lowman W Laux LC Millichap JJ The ketogenic diet in children 3 years of age or younger: a 10-year single-center experience. Sci Rep. (2019) 9:8736. 10.1038/s41598-019-45147-6 31217425 PMC6584655

[17] Bergqvist AC Schall JI Gallagher PR Cnaan A Stallings VA. Fasting versus gradual initiation of the ketogenic diet: a prospective, randomized clinical trial of efficacy. Epilepsia. (2005) 46:1810–9. 10.1111/j.1528-1167.2005.00282.x 16302862

[18] Kossoff EH Pyzik PL Furth SL Hladky HD Freeman JM Vining EP. Kidney stones, carbonic anhydrase inhibitors, and the ketogenic diet. Epilepsia. (2002) 43:1168–71. 10.1046/j.1528-1157.2002.11302.x 12366731

[19] Chesney D Brouhard BH Wyllie E Powaski K. Biochemical abnormalities of the ketogenic diet in children. Clin Pediatr. (1999) 38:107–9. 10.1177/000992289903800207 10047944

[20] Nassar MF El-Rashidy OF Abdelhamed MH Shata MO. Modified Atkins diet for drug-resistant epilepsy and the risk of urolithiasis. Pediatr Res. (2022) 91:149–53. 10.1038/s41390-021-01732-y 34497357

[21] Kang HC Chung DE Kim DW Kim HD. Early-and late-onset complications of the ketogenic diet for intractable epilepsy. Epilepsia. (2004) 45:1116–23. 10.1111/j.0013-9580.2004.10004.x 15329077

[22] Kossoff EH Pyzik PL Rubenstein JE Christina Bergqvist AG Buchhalter JR Donner EJ Combined ketogenic diet and vagus nerve stimulation: rational polytherapy? Epilepsia. (2007) 48:77–81. 10.1111/j.1528-1167.2006.00903.xCITE 17241211

[23] Zarnowska IM. Therapeutic use of the ketogenic diet in refractory epilepsy: what we know and what still needs to be learned. Nutrients. (2020) 12:2616. 10.3390/nu12092616 32867258 PMC7551948

[24] Martin-McGill KJ Lambert B Whiteley VJ Wood S Neal EG Simpson ZR Ketogenic dietitians research network (KDRN). Understanding the core principles of a 'modified ketogenic diet': a UK and Ireland perspective. J Hum Nutr Diet. (2019) 32(3):385–90. 10.1111/jhn.12637 30859652

[25] Butte NF. 1.3. 2 energy requirements of infants, children and adolescents. In: Koletzko B, editors. Pediatric nutrition in practice 2nd revised edition. Munich: Karger Publishers; (2015), p. 34–40.; pp. 34–40.

[26] Butte NF. 1.3.2 Energy requirements of infants, children and adolescents. 1.3 Nutritional needs. World Rev Nutr Diet. (2015) 113:34–40. 10.1159/000360315 25906853

[27] Drewnowski A Rehm CD Constant F. Water and beverage consumption among children age 4-13y in the United States: analyses of 2005–2010 NHANES data. Nutr J. (2013) 12:85. 10.1186/1475-2891-12-85 23782914 PMC3698018

[28] El-Rashidy OF Youssef MM Elgendy YG Mohsen MA Morsy SM Dawh SA Selenium and antioxidant levels in children with intractable epilepsy receiving ketogenic diet. Acta Neurol Belg. (2020) 120:375–80. 10.1007/s13760-020-01310-9 32107714

[29] Kossoff EH Laux LC Blackford R Morrison PF Pyzik PL Hamdy RM When do seizures usually improve with the ketogenic diet? Epilepsia. (2008) 49:329–33. 10.1111/j.1528-1167.2007.01417.x 18028405

[30] Kossoff EH Turner Z Adams J Bessone SK Avallone J McDonald TJ Ketogenic diet therapy provision in the COVID-19 pandemic: dual-center experience and recommendations. Epilepsy Behav. (2020) 111:107181. 10.1016/j.yebeh.2020.107181 32512472 PMC7247448

[31] Loftus TJ Tighe PJ Ozrazgat-Baslanti T Davis JP Ruppert MM Ren Y Ideal algorithms in healthcare: explainable, dynamic, precise, autonomous, fair, and reproducible. PLOS Digit Health. (2022) 1(1):e0000006. 10.1371/journal.pdig.0000006 36532301 PMC9754299

[32] Meira I D Romão TT Pires do Prado HJ Krüger LT Pires MEP da Conceição PO. Ketogenic diet and epilepsy: what we know so far. Front Neurosci. (2019) 13:5. 10.3389/2Ffnins.2019.00005 30760973 PMC6361831

[33] Armeno M Caraballo R. The evolving indications of KD therapy. Epilepsy Res. (2020) 163:106340. 10.1016/j.eplepsyres.2020.106340 32330835

[34] Kossoff EH Turner Z Bergey GK. Home-guided use of the ketogenic diet in a patient for more than 20 years. Pediatr Neurol. (2007) 36(6):424–5. 10.1016/j.pediatrneurol.2007.01.013 17560509

[35] McGaugh E Barthel B. A review of ketogenic diet and lifestyle. Mo Med. (2022) 119(1):84–88. PMID: . 36033148 PMC9312449

[36] Williams TJ Cervenka MC. The role for ketogenic diets in epilepsy and status epilepticus in adults. Clin Neurophysiol Pract. (2017) 2:154–60. 10.1016/j.cnp.2017.06.001 30214989 PMC6123874

