# Supplementary material for: Ketogenic diet in childhood epilepsy: clinical algorithm in a tertiary care center
Source: Front Pediatr. 2023 Jul 6;11:1221781. doi: 10.3389/fped.2023.1221781 (PMC10361660; doi:10.3389/fped.2023.1221781)
Supplement: Supplementary file 1 [file Datasheet1.pdf]

Appendix A

Potential Complications Arising from the KD and Their Management

If a patient fell sick during the treatment, the KD was demoted as compared to the treatment of the patient's illness. However, sugar-free medication was encouraged whenever possible to avoid loss of ketosis. The major complications that could arise due to the KD have been listed below, along with their management.

[1] Vomiting and Diarrhea

When faced with vomiting and diarrhea, the KD was stopped, and fluids (low in carbohydrates) were administered. Low glucose oral rehydration fluids were diluted in the ratio of 2:1 to prevent an upset in ketosis. Intravenous fluids, at an appropriate strength, were used [1].

Blood glucose measurements were taken throughout the day (at any time of fasting), and the patient was observed for signs of hypoglycemia. Regular ketone monitoring was performed to pre-empt the possibility of hyper ketosis. Hyper ketosis or hypoglycemia was treated quickly by administering 20–30 mL of a 10% carbohydrate-containing drink. The offending levels were rechecked after 15 minutes, and the drink administration was repeated if necessary [2, 3].

Once the vomiting had subsided, the KD was reintroduced gradually with half the usual meal quantities for the first 24–48 h, or as per tolerance. The amount of fat in the diet was reduced temporarily (24–48 h) and increased gradually as tolerated. For tube-fed patients, half-strength feeds were given for 24–48 h and gradually built up to full strength as tolerated [2].

In case of infections (colds and influenza), using sugar-free paracetamol or suppositories was encouraged. Certain infections may cause a drop in ketones but once recovered, ketosis should gradually return [2].

[2] Hyper ketosis

This condition is where the ketone levels become too high and may occur after starting the diet or during an illness. Symptoms include rapid panting/breathing, increased heart rate, facial flushing, irritability, vomiting, and unexpected lethargy.

Hyper ketosis was confirmed by checking the urine sample with Ketostix®. A high level of urinary ketones (acetoacetate) would be indicated by an immediate deep purple color with a titer of ≥3–4. In addition, blood ketones were checked via monitor with high levels of >6 mmol/L.

This condition was managed by administering 30 mL pure fruit juice (orange juice). After 15–20 minutes, the levels were rechecked, and additional juice was administered as required [20]. Alteration of diet parameters was considered if the condition was persistent, and the patient was symptomatic.

[3] Hypoglycemia

This condition arises when the blood glucose levels drop to a dangerous level. We start interfering if blood glucose is <2.5 mmol/L (45 mg/dL). Treatment of hypoglycemia using rapidly absorbed carbohydrates (like orange juice) is the simplest form of combatting the situation [3]. Severe symptomatic hypoglycemia was treated with 2 mL/Kg of 10% dextrose, followed by an infusion of 2.5% or 5% dextrose.

[4] Metabolic Acidosis

Metabolic acidosis occurs when there is either an increase in the production of acids or a loss of bicarbonate from the body, which overwhelms the mechanisms of homeostasis [4]. It is known that children on the KD have lower serum bicarbonate. However, certain patients are in the risk zone, namely those who take topiramate, zonisamide, or acetozalomide, and those who have renal impairment [1].

Symptoms of this condition include increased seizures, clamminess and pale skin, confusion, and Kussmaul breathing (increased rate and depth of breathing).

Confirmation was obtained by checking electrolytes, bicarbonate, urinary ketones, blood ketones (β-hydroxybutyrate), and blood gas levels. We start interfering if blood ketones are > 6 mmol/L. The management involved adequate hydration with water or sugar-free oral fluids, normal saline (0.9% NaCl) in IV if required, reduction/withdrawal of aforementioned medications on the recommendation of the Keto team, or diet manipulation [1, 5].

1. Kim SH, Shaw A, Blackford R, Lowman W, Laux LC, Millichap JJ, Nordli Jr DR. The ketogenic diet in children 3 years of age or younger: a 10-year single-center experience. Sci Rep 2019; 9:8736. https://doi.org/10.1038/s41598-019-45147-6 16
2. Hartman AL, Vining EP. Clinical aspects of the ketogenic diet. Epilepsia 2007; 48:31-42. https://doi.org/10.1111/j.1528-1167.2007.00914.x 14

3. Nassar MF, El-Rashidy OF, Abdelhamed MH, Shata MO. Modified Atkins diet for drug-resistant epilepsy and the risk of urolithiasis. Pediatr Res 2022; 91:149-53. https://doi.org/10.1038/s41390-021-01732-y 20
4. Sampath A, Kossoff EH, Furth SL, Pyzik PL, Vining EP. Kidney stones and the ketogenic diet: risk factors and prevention. J Child Neurol 2007; 22: 375-8. https://doi.org/10.1177/0883073807301926
5. Kraut JA, Madias NE. Metabolic acidosis: pathophysiology, diagnosis and management. Nat Rev Nephrol 2010; 6: 274-85. https://doi.org/10.1038/nrneph.2010.33
